# A perceptual aid to delineating the extent of potential mammographic abnormalities

**DOI:** 10.1186/bcr3781

**Published:** 2015-11-05

**Authors:** Arul Selvan, Yan Chen, Alastair Gale

**Affiliations:** 1 Sheffield Hallam University, Sheffield, UK; 2 Loughborough University, Loughborough, UK

## Poster presentation

Being able to accurately determine the extent of a possible malignancy on a mammogram is an important task as this can affect the potential follow-up surgical treatment that a woman receives after breast screening. It is known that this can be a difficult task, particularly where the lesion has diffuse abnormalities. A potential computer-aided approach is to employ hierarchical clustering-based segmentation (HCS) and this interactive educational exhibit dynamically demonstrates this technique. HCS is an unsupervised segmentation process that when applied to an image yields a hierarchy of segmentations based on image pixel dissimilarities and so can be used to highlight areas in the mammographic image to aid interpretation.

A set of 15 known difficult FFDM mammographic cases were selected from PERFORMS case sets where expert radiologists had previously delineated the extent of various abnormalities. Regions of interest (ROI) around these abnormalities were extracted from the DICOM images and processed using HCS algorithms resulting in a set of paired original mammographic ROI images and related HCS processed ROI images. In the exhibit these paired images are presented and delegates interactively select which of the pair they think best identifies abnormality extent. The original expert delineated abnormality is then provided as feedback. Over the course of the conference, data will be collected on how useful the HCS approach has been found and this information fed back to participants. The learning objectives are to demonstrate the potential of this approach in increasing the perceptual recognition of abnormal appearances.

